# Functional impact of bilateral vestibular loss and the unexplained complaint of oscillopsia

**DOI:** 10.3389/fneur.2024.1365369

**Published:** 2024-04-22

**Authors:** Dario Geisinger, Zohar Elyoseph, Roy Zaltzman, Matti Mintz, Carlos R. Gordon

**Affiliations:** ^1^Faculty of Medical and Health Sciences, Tel Aviv University, Tel Aviv, Israel; ^2^Department of Psychology and Educational Counseling, The Center for Psychobiological Research, Max Stern Yezreel Valley College, Yezreel Valley, Israel; ^3^School of Psychological Sciences, Tel Aviv University, Tel Aviv, Israel; ^4^Department of Brain Sciences, Faculty of Medicine, Imperial College London, London, United Kingdom; ^5^Department of Neurology, Meir Medical Center, Kfar Saba, Israel; ^6^Sagol School of Neuroscience, Tel Aviv University, Tel Aviv, Israel

**Keywords:** bilateral vestibular areflexia, oscillopsia, Machado–Joseph disease, peripheral vestibular and central disorders, saccades, spatial orientation

## Abstract

**Introduction:**

The vestibulo-ocular reflex (VOR) stabilizes vision during head movements. VOR disorders lead to symptoms such as imbalance, dizziness, and oscillopsia. Despite similar VOR dysfunction, patients display diverse complaints. This study analyses saccades, balance, and spatial orientation in chronic peripheral and central VOR disorders, specifically examining the impact of oscillopsia.

**Methods:**

Participants involved 15 patients with peripheral bilateral vestibular loss (pBVL), 21 patients with clinically and genetically confirmed Machado–Joseph disease (MJD) who also have bilateral vestibular deficit, and 22 healthy controls. All pBVL and MJD participants were tested at least 9 months after the onset of symptoms and underwent a detailed clinical neuro-otological evaluation at the Dizziness and Eye Movements Clinic of the Meir Medical Center.

**Results:**

Among the 15 patients with pBVL and 21 patients with MJD, only 5 patients with pBVL complained of chronic oscillopsia while none of the patients with MJD reported this complaint. Comparison between groups exhibited significant differences in vestibular, eye movements, balance, and spatial orientation. When comparing oscillopsia with no-oscillopsia subjects, significant differences were found in the dynamic visual acuity test, the saccade latency of eye movements, and the triangle completion test.

**Discussion:**

Even though there is a significant VOR gain impairment in MJD with some subjects having less VOR gain than pBVL with reported oscillopsia, no individuals with MJD reported experiencing oscillopsia. This study further supports that subjects experiencing oscillopsia present a real impairment to stabilize the image on the retina, whereas those without oscillopsia may utilize saccade strategies to cope with it and may also rely on visual information for spatial orientation. Finding objective differences will help to understand the causes of the oscillopsia experience and develop coping strategies to overcome it.

## Introduction

The vestibulo-ocular reflex (VOR) is a fast and precise reflex that maintains a stable gaze in space during abrupt head movements and in this way affords a focused vision of the surrounding environment. This aspect is achieved by aiming for a VOR gain [defined as the eye velocity divided by the head velocity (1)] close to 1, maintaining the image stable on the retina. This phenomenon explains why a normal person can walk, jump, or travel in a vehicle while perceiving the world as stable during movement. When the VOR gain is too low, the eyes are unable to compensate for the head movements and the image in the retina suffers from retinal slip. The primary long-lasting complaints in vestibular, peripheral, or central disorders with VOR impairment include imbalance, dizziness, blurred vision, or oscillopsia during head movements (2). Imbalance can be worsened in darkness or on uneven grounds. Dizziness is an ill-defined sensation encompassing a wide variety of feelings such as light-headedness, giddiness, unsteadiness, weaving, swaying, and floating. Blurred vision refers to the loss of sharpness in vision and the inability to see fine details. Oscillopsia is a rare complaint of an illusion of an unstable or jumpy visual world, particularly noticeable during head movement, such as walking.

In a clinical practice, it is common to encounter chronic patients with similar bilateral VOR impairment but present different levels of complaints. This discrepancy has been partially explained by the different levels of compensation. It has been suggested that patients can achieve compensation using different strategies of adaptation (changing the gain of the VOR) or reweighting non-vestibular sensory input, using different mechanisms such as the cervical ocular response, central compensation via new synapse formation, cortical reorganization, recovery of damaged hair cells, behavioral adaptation (3), and saccadic eye movement strategies (4). Moreover, some studies suggested that the presence vs. absence of oscillopsia complaints is related to emotional and other psychological factors (5). In this context, the most extreme example is probably the lack of correlation between the VOR loss and the complaints of oscillopsia. For example, some patients with almost complete VOR loss do not necessarily complain about oscillopsia.

Bilateral vestibular loss (BVL) sometimes referred to as bilateral vestibulopathy (6) or bilateral vestibular weakness is a relatively rare condition with the majority of cases having unknown etiology or being related to aminoglycoside ototoxicity (3). The most common clinical presentation of acute BVL is oscillopsia and imbalance with symptoms improving with vestibular rehabilitation. However, there is a small percentage of subjects that complain of chronic oscillopsia even after 16 years after the acute event (5).

Spinocerebellar ataxia type 3 (SCA3), also known as Machado–Joseph disease (MJD), is the most frequent hereditary SCA (7). Among other eye movement abnormalities (8), MJD causes significant VOR deficit (9–14). Although MJD subjects present with bilateral vestibular deficit, complaints of oscillopsia are rare and mostly related to the presence of nystagmus (9, 15).

The present study provides an analysis based on quantifying the vestibular function to explore the characteristics of saccades, balance, gaze stability, and spatial orientation in chronic peripheral and central BVL, with a particular emphasis on how these elements are expressed in the presence or absence of oscillopsia in an attempt to identify potential factors that may aid in the management of oscillopsia.

## Materials and methods

### Participants

The participants included 15 individuals with peripheral BVL (pBVL, 9 women, 6 men; age 60 ± 13 years), 21 with clinically and genetically confirmed MJD (15 women, 6 men; age 58 ± 15 years), and 22 healthy controls (9 women, 13 men; age 56 ± 14 years), the latter having no history of neurological, sensory, or balance problems. All pBVL and MJD participants were tested at least 9 months after the onset of symptoms. All participants underwent a detailed clinical neuro-otological evaluation at the Dizziness and Eye Movements Clinic of the Meir Medical Center. To determine the presence of oscillopsia in both pBVL and MJD groups, the following questions were asked: Does the world around you seem to move or jump when you walk or move? Is it stronger when you walk or move than when your head is static? When the answer was “Yes” to both questions, we categorized the subject as having complaints of oscillopsia.

The study protocol was approved by the Ethics Committee (Institutional Review Board) of the Meir Medical Center, Kfar Saba, Israel, and followed the tenets of the Declaration of Helsinki. The study was also reviewed and approved by the Ethics Committee of Tel Aviv University, Israel. All participants signed an informed consent form after receiving an explanation regarding the research procedures.

### Experimental tests

#### Vestibular tests

##### Video head impulse test (vHIT)

vHIT was used to examine the angular VOR for all semicircular canals and determine the compensatory saccades pattern. Participants were instructed to fixate on a central point on a screen located 150 cm ahead. The examiner executed rapid and unpredictable angular rotations of the participant’s head in the horizontal plane and diagonally in the left anterior–right posterior (LARP) and right anterior–left posterior (RALP) planes (16, 17). VOR gain (eye velocity/head velocity) together with the level of saccade grouping (PR score) and VOR relative gain asymmetry per canal for BVL was obtained from the head and eye movements (ICS, Otometrics).^1^ The PR score is an indicator of how scattered the catch-up saccades are, being 0 minimum scattered and 100 maximum scattered catch-up saccades. Scattered saccades are observed when the corrective saccades’ latencies are spread along the response, while grouped saccades are observed time-locked to the start of the head movement (18). Covert saccades are corrective saccades that occur while the head is still rotating while overt saccades appear after the head rotation. The presence of these saccades is determined for each canal as a percentage over all trials. The test at each plane was repeated 10 times.

##### Suppression head impulse test (SHIMP)

The SHIMP variant (ICS, Otometrics) was used to examine the remaining angular horizontal vestibular function together with the compensatory and anti-compensatory saccade patterns. Participants were instructed to track a light point generated using a head-fixed laser. The examiner executed rapid and unpredictable angular rotations of the participant’s head in the horizontal plane at a velocity that was fast enough to ensure the presence of VOR response before the VOR cancellation saccade could be activated (~80 ms). In a normal case, we expect that reaching the moving target will be accomplished by a saccade ipsilateral to the head movement following a contralateral VOR response. SHIMP is assumed to reflect the remaining VOR function by means of the SHIMP gain (eye velocity/head velocity) (19). The PR score was also obtained for SHIMP. The test toward each side was repeated 10 times.

##### Cervical vestibular evoked myogenic potential (cVEMP)

cVEMP was used to test the saccular function by recording the relaxation of a tonically contracted sternocleidomastoid muscle (SCM) in response to a tone stimulus, mediated by the vestibulo-colic reflex pathway (20). cVEMPs are recorded by placing the active electrode on the middle third of the SCM, the reference electrode 2 cm below the mid-point between the right and left junctions of the clavicle and the SCM, and the ground electrode on the forehead of the participant. cVEMPs were obtained using a Bio-Logic System (NavigatorPRO BIOLOGIC, Natus, Germany) with the binaural short tone bursts of 95 dB nHL (500 Hz, 5 cycles/s, 2 ms rise/fall, and plateau time = 0) applied through insert earphones. Participants were instructed to raise their head straight ahead from a supine position until the operator verified a reliable SCM contraction. In total, 200 stimuli were averaged to obtain the P13 and N23 components of the cVEMP response. In a quest to validate a case of absent cVEMP response, the procedure was repeated with the head rotated to the contralateral side of the unilateral recording if the previous method showed inconclusive results to avoid false-negative results (21).

##### Dynamic visual acuity (DVA)

The DVA test was used to determine the contribution of horizontal head movements to the visual acuity of pBVL subjects. The test requires the operator to move the patient’s head with a rotation frequency of approximately 2 Hz while the subject identifies an icon in a Snellen 3-m eye chart (22). The impact of the VOR impairment on the ability to stabilize the image in the retina is determined by the decrease in visual acuity during head movement. The difference in rows between static (test performed without head movements) and dynamic visual acuity was used as the DVA outcome, setting half values for a line where at least half of the icons were correctly identified.

##### Subjective visual vertical (SVV)

This computerized test mainly measures the functioning of the utricle (23). The patient is asked to align a tilted line on a computer screen by means of a controller, with the vertical axis. The procedure is done in a completely dark room with the subject looking at the computer screen through a tube attached to the screen in order to eliminate any external spatial references. Once the subject reports that a line is aligned with the vertical, a new tilted line appears on the screen and the process is repeated. The average deviation from the true vertical axis is calculated for both clockwise and counterclockwise directions (24). The test was conducted on a black background (SVV) and a rotating pattern of random dots (dynSVV). We used “Rod and Disk” software^2^ available from the Neuro-Otology Unit of the Imperial College London and calculated the average absolute deviation in degree (25).

#### Eye movement test

##### Saccades test

The Saccades test was used to determine the dynamics of the saccade eye movements. This test involves sitting the patient at a fixed distance from a white screen and asking the patient to follow the laser dot that is projected from a pair of goggles while measuring the eye movements (ICS, Otometrics). The saccade amplitude presented varied randomly between 7.5 and 15 degrees. However, a custom analysis of the saccades was conducted and not the standard results provided by the system. For each trial (between 30 s and 1 min of saccades), only the 7.5-degree amplitude saccade was used. Latency and Velocity values were provided by the system, and an Accuracy error variable was calculated from the raw data file as the absolute error in percentage from the expected amplitude. The rationale for this Accuracy error variable is that the system provides by default an accuracy value that is a percentage of the expected accuracy. When there is an undershoot saccade, the value will be less than 100%; when the saccade amplitude coincides with the target, the value is 100% and overshoot saccades have values over 100%. In this case, when comparing mean Accuracy, the actual error is compensated by undershoot and overshoot saccades. This aspect may produce results that reflect mean accuracy but do not reflect the error on the saccade. Our analysis simply takes the absolute value of the difference between the expected and actual saccade as a percentage of the expected amplitude of the saccade, emphasizing the error at the expense of distinguishing between undershoot and overshoot saccades. As the test by design presents much more 7.5-degree saccades than 15-degree large saccades, these large saccades were very low in number and did not provide enough data for an average to be reliable.

#### Balance

##### Posturography

This test is a computerized test of body sway by means of a force platform (Bertec, USA). Body sway is recorded while subjects perform the four conditions of the modified Clinical Test of Sensory Integration in Balance (mCTSIB), which consists of standing still on condition 1: eyes open over the firm surface (EO); condition 2: eyes closed over the firm surface (EC); condition 3: eyes open over foam (EOF); and condition 4: eyes closed over foam (ECF) (26). The stability score obtained from Bertec Workgroup software was used as an indicator of balance.

#### Spatial orientation tests

##### The triangle completion task (TCt)

This test measures path integration and navigation (27). The experimenter leads the patient from a starting point on a path consisting of two sides of a right triangle and asks her/him to return from the stopping point to the starting point (complete triangle) with eyes closed. From the starting point, the subject walks forward 1 or 2 m, turns right or left, walks 1 or 2 m, and is then instructed to return to the start position. The actual order of the eight combinations of distance and sides was random, and all subjects performed all triangles. The parameters used for this analysis were angle error (Angle) calculated as the mean of the absolute values of the difference between the correct angle rotation from the stopping point (straight line to the start point) and the actual angle rotation performed, distance error (Distance) calculated as the mean of the absolute values of the difference between the required travel distance from the stopping point to the starting point and the actual traveled distance, and distance of deviation (Deviation) defined as the distance from the starting point to the point where the subject reached when completing the triangle. This test takes approximately 15 min to complete.

##### Object perspective taking test (OPTt)

This test measures spatial perspective taking, the ability of an individual to view a situation from a different spatial perspective (28). Subjects are presented with a paper with pictures of an array of seven objects and an “arrow circle.” Subjects imagine that they stand on a first object facing in the direction of a second object. Relative to their position, they are required to indicate the direction of a third object and mark it on the arrow circle. The average judgment error is calculated for the absolute angular deviations across the 12 cases of the test.

##### Spatial reasoning test (SRt)

This test refers to the capacity to think about objects in three dimensions and to understand how an object would look like when rotated.^3^ The subject is presented with a series of unfolded cubes and four different folded cubes for each unfolded one and asked to mark which cube cannot be made based on the unfolded one. This test takes approximately 15 min to complete.

### Statistical analysis

Mixed ANOVA with groups (Control, pBVL, and MJD) as independent between variables was used to compare vHIT (six canals as independent within variable), SHIMP (two sides as independent within variable), SVV (background condition as independent within variable), and posturography (test conditions as independent within variable). Repeated-measures ANOVA for each group and one-way ANOVA were used as *post-hoc* tests.

MANOVA with groups as independent between-subjects variables was used to compare saccades (Velocity, Amplitude and Latency as dependent variables) and TCt (Angle, Distance and Deviation as dependent variables).

One-way ANOVA with groups as independent variables was used to compare OPTt and SRt. The Mann–Whitney U test was used in all comparisons between oscillopsia and no oscillopsia in the BVL group, and the Pearson correlation test was used to test whether distance and deviation are related. The chi-square test was used to compare the proportions of the present cVEMP response between groups. In an attempt to identify a potential physiological discriminator between subjects presenting oscillopsia and those without the complaint, receiving operating characteristic (ROC) curve was used to determine the quality of a classifier. In the case that a subject did not perform a test, the number of subjects that conducted the test is specified in the results.

## Results

Among the 15 patients with pBVL and 21 patients with MJD, only 5 patients with pBVL complained about chronic oscillopsia, while none of the MJD participants reported this complaint.

### Vestibular tests

**Figure 1:**
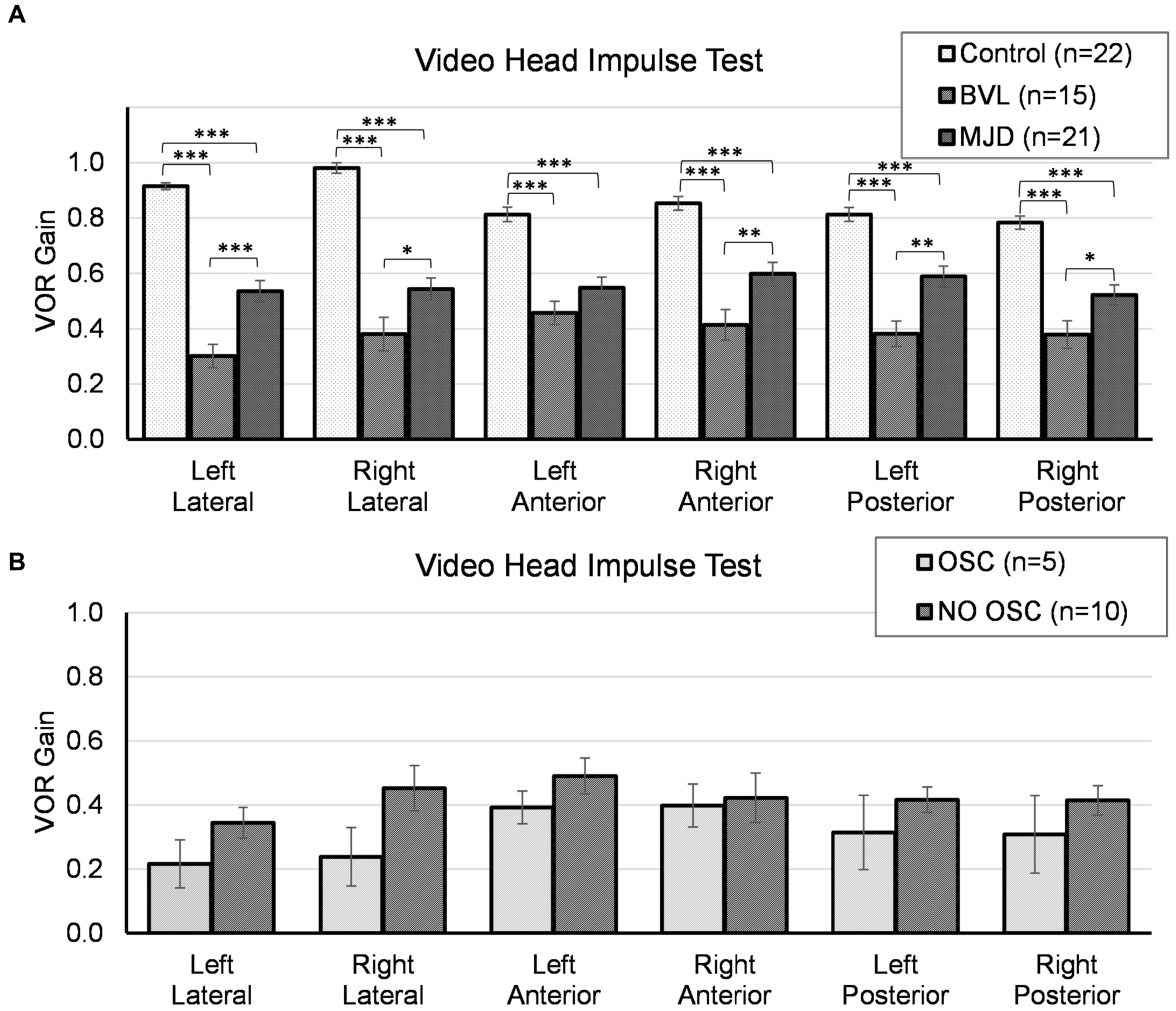
Video head impulse test for all canals in **(A)** Control, BVL, and MJD groups, and **(B)** the BVL group with oscillopsia and without oscillopsia. *p < 0.05, **p < 0.01, ***p < 0.001.

The lowest VOR gain was found in pBVL followed by MJD and Control populations for all canals, except LA between MJD and pBVL, as shown in Figure 1A. The analysis of VOR gain on vHIT showed a significant main effect of Groups [*F*(2, 55) = 1467.97, *p* < 0.001], Canals [*F*(3.84, 211.48) = 2.93, *p* < 0.05], and Groups-by-Canals interaction [*F*(7.69, 211.48) = 5.09, *p* < 0.001]. Between-groups comparisons confirmed that there are significant differences for all canals between Control, MJD, and pBVL groups (*p* < 0.01 in all six cases). Except for LA between MJD and pBVL, all canals presented significant differences between Groups. The *post-hoc* test showed that Control [*F*(5, 105) = 14.84, *p* < 0.001] and pBVL [*F*(5, 70) = 2.64, *p* = 0.030] but not MJD [*F*(3.29, 65.77) = 1.12, *p* = 0.35] had differences between the canals within each group. Comparison within the pBVL group of patients with and without oscillopsia (see Figure 1B) found no significant differences in VOR gain between both groups in all six canals (*p* > 0.05 in all six cases) and no significant differences in relative gain asymmetry for lateral canal (*p* = 0.859), anterior canal (*p* = 0.165), or posterior canal (*p* = 0.953). BVL subjects made either only overt saccades (*n* = 4) or a mixture of both covert and overt saccades (*n* = 11), while no subject did only covert saccades. No differences were found in the percentage of covert or overt saccades between BVL with and without oscillopsia (covert *p* = 0.95; overt *p* = 1), and also, no differences were found in the PR score (*p* = 0.371).

**Figure 2:**
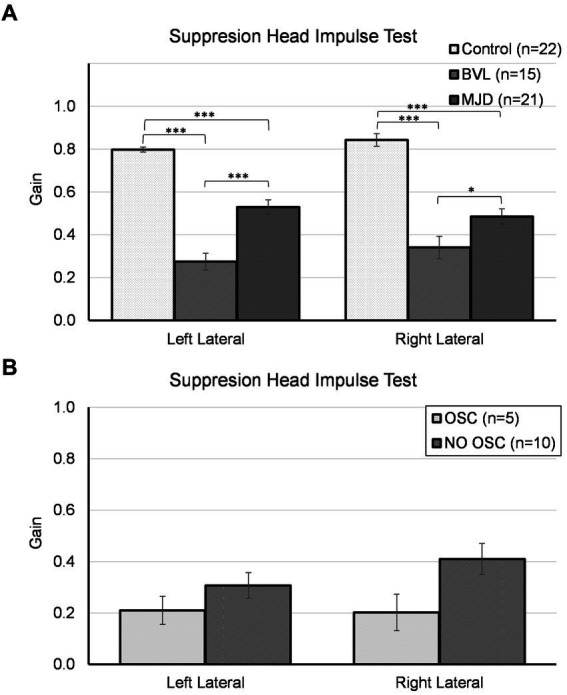
Suppression head impulse test for horizontal canals in **(A)** the Control, BVL, and MJD groups, and **(B)** the BVL group with oscillopsia and without oscillopsia.

The lowest SHIMP gain was found in pBVL followed by MJD and Control populations for both horizontal canals as shown in Figure 2. The analysis of SHIMP gain showed a significant main effect of Groups [*F*(2, 55) = 68.25, *p* < 0.001], no main effect of sides [*F*(1, 55) = 2.09, *p* = 0.15], and significant Groups-by-side interaction [*F*(2, 55) = 4.95, *p* = 0.011]. Between-groups comparisons confirmed that there are significant differences in both left and right sides among Control, MJD, and pBVL (*p* < 0.001 for both sides) groups having higher SHIMP gain in Control than in pBVL (*p* < 0.001 for both right and left sides) and MJD (*p* < 0.001 for both right and left sides) groups and higher SHIMP gain in MJD than in pBVL (*p* < 0.001 for left side; *p* = 0.038 for right side) group. The *post-hoc* test showed no differences between sides within the Control, pBVL, and MJD groups (*p* = 0.075, *p* = 0.085, and *p* = 0.056 respectively). Comparison within the pBVL group of patients with and without oscillopsia found no significant differences between the groups in both left and right lateral canals (*p* > 0.05 in both cases). Peripheral BVL subjects made either only overt saccades (*n* = 14) or a mixture of both covert and overt saccades (*n* = 1, in one ear only), while no subject did only covert saccades. No differences were found in the percentage of covert or overt saccades between pBVL with and without oscillopsia (covert *p* = 0.77, overt *p* = 0.17), and also, no differences were found in the PR score (*p* = 0.59).

**Figure 3:**
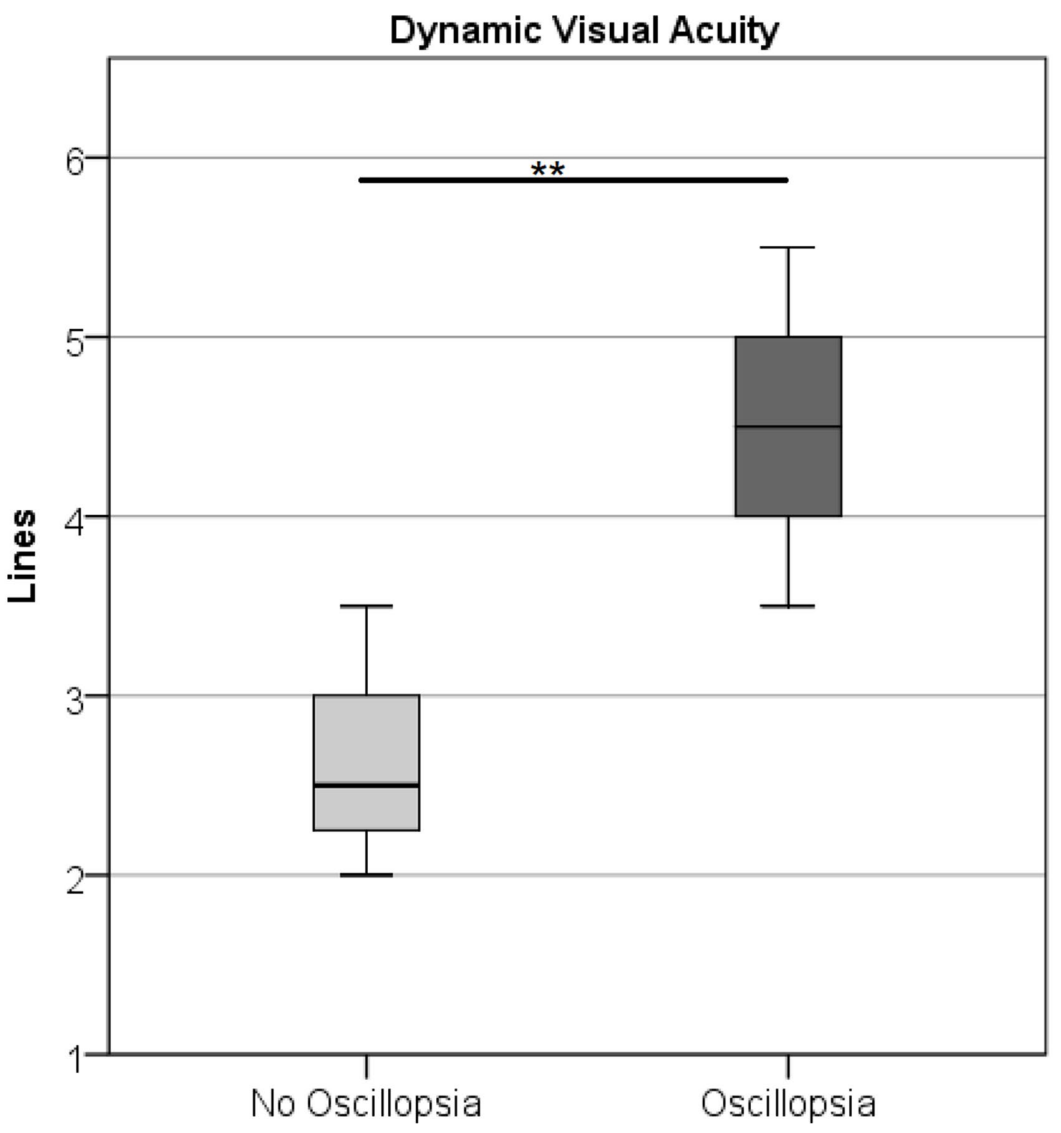
Dynamic visual acuity in the BVL group without and with oscillopsia displaying mean (○) and CI at 95%. Oscillopsia group 4.5 missed lines ± 0.79 and no-oscillopsia group 2.6 missed lines ± 0.56 (mean ± STD), *p* < 0.01.

DVA was higher (U = 0.5, Z = −2.78, *p* < 0.01) in the oscillopsia group (*n* = 5) than in the no-oscillopsia group (*n* = 7) as shown in Figure 3.

The SVV analysis showed no main effect of Group [*F*(2, 50) = 2.364, *p* = 0.104], the significant main effect of background [*F*(2, 100) = 76.81, *p* < 0.001], and no Groups-by-background interaction [*F*(4, 100) = 1.602, *p* = 0.180]. Pairwise comparison showed SVV with a static background was significantly lower than with a rotating background (*p* < 0.001 for both rotation directions), but no difference was found between the two rotation directions (*p* = 1). Comparison within the pBVL group of patients with and without oscillopsia found no significant differences in stability (*p* > 0.05) or the two rotating background conditions (*p* > 0.05 in both cases).

The cVEMP response was absent in 69, 20, and 19% of the participants in pBVL, MJD, and Control groups, respectively, and the predominant absence of the responses in the BVL was significant [χ2 (2, N = 54) = 11.31, *p* < 0.01]. No difference was found between oscillopsia and no oscillopsia using Fisher’s exact test (*p* > 0.05).

### Eye movements

**Figure 4:**
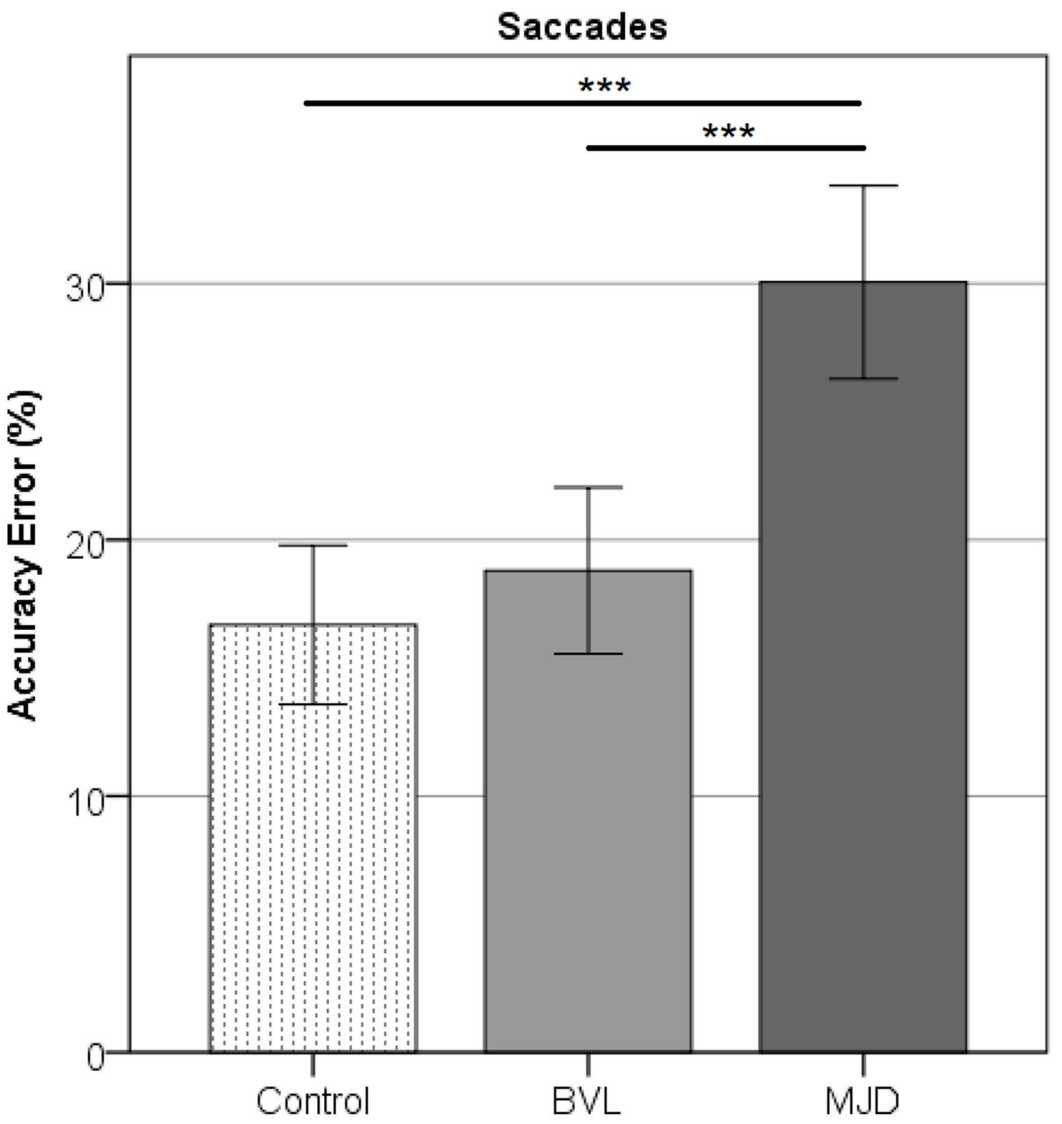
Accuracy error in horizontal saccades showing higher error for MJD as compared to the Control and pBVL groups (*p* < 0.001 in both cases).

**Figure 5:**
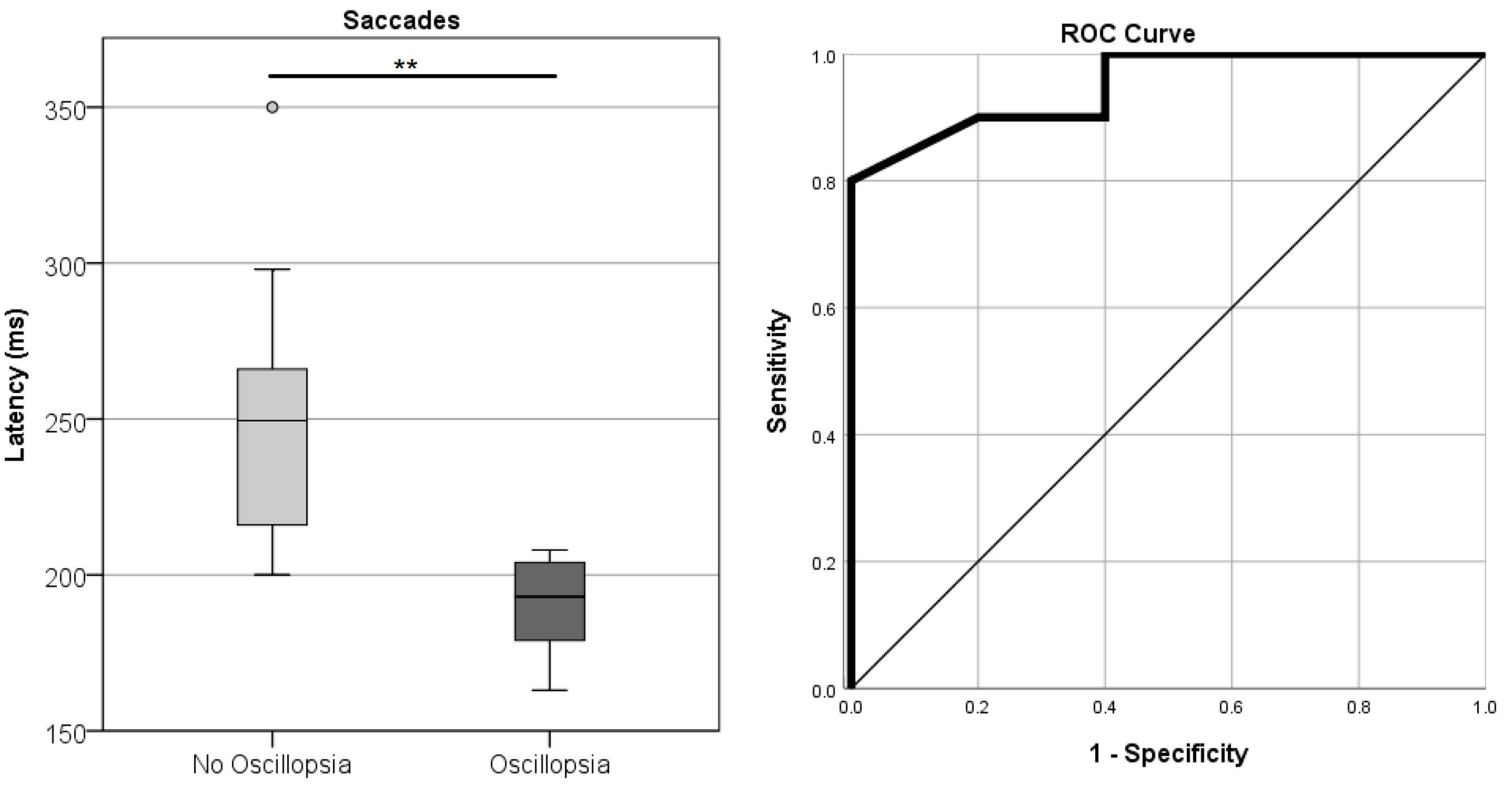
Latency of horizontal saccades in the BVL group without and with oscillopsia together with the ROC curve plot.

Saccade Accuracy error was found to be higher in MJD than in both Control and BVL groups (see Figure 4 and Table 1). There was a significant difference in saccade characteristics between groups [Wilks’ Lambda = 0.50, *F*(7.44, 53) = 1783, *p* < 0.001] for Accuracy error [*F*(2, 55) = 18.6, *p* < 0.001] but not for Velocity [*F*(2, 55) = 1.36, *p* = 0.27] or Latency [*F*(2, 55) = 2.39, *p* = 0.101]. The post-hoc test found higher Accuracy errors in MJD than in Control and BVL groups (*p* < 0.001 in all cases). Comparison within the BVL groups of patients with and without oscillopsia found that the Latency of the oscillopsia group was significantly lower than that in the non-oscillopsia group (U = 2.5, Z = −2.76, *p* < 0.01). The ROC analysis probed Latency to be an excellent classifier between oscillopsia and non-oscillopsia with an area under the curve (AUC = 0.950) as shown in Figure 5. No significant differences were found between the groups in Velocity and Accuracy (*p* = 0.16, *p* = 0.44 respectively).

**Table 1:** Reflexive saccades.

	Accuracy error (%)	Latency (ms)	Velocity (deg/s)
Control	16.7 ± 1.62	208 ± 9.2	250 ± 9.5
MJD	30.0 ± 1.66	234 ± 9.4	268 ± 9.8
pBVL	18.8 ± 1.96	232 ± 11	245 ± 12

Data represent mean ± SEM.

### Balance

**Figure 6:**
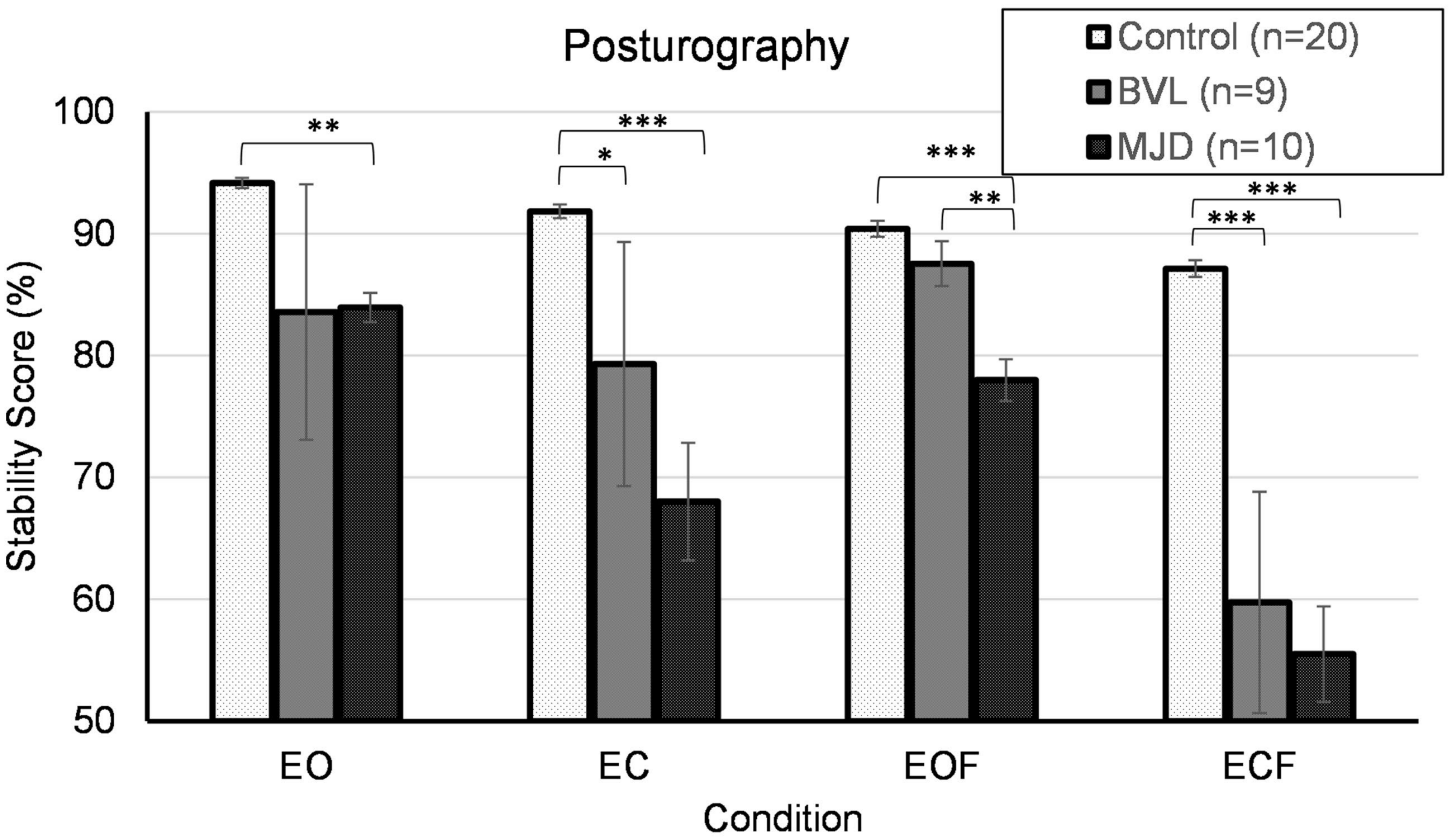
Posturography. EO, eyes open; EC, eyes closed; EOF, eyes open foam; ECF, eyes closed foam.

Figure 6 shows that Controls group (*n* = 20) had better stability scores than BVL (*n* = 9) and MJD (*n* = 10) groups. The stability score measured using posturography showed a significant main effect of 4ups [*F*(2, 36) = 13.90, *p* < 0.001], significant main effect of standing condition [*F*(2.13, 76.58) = 27.18, *p* < 0.001], and significant Groups-by-condition interaction [*F*(4.25, 76.58) = 5.30, *p* < 0.001]. The *post-hoc* tests further show significant difference between groups in all conditions (EO *p* < 0.01, EC *p* < 0.001, EOF *p* < 0.001, ECF *p* < 0.001) with differences in EO between Control and MJD (*p* < 0.01) groups, in EC between Control and BVL (*p* < 0.05) and between Control and MJD (*p* < 0.001) groups, in EOF between Control and MJD (*p* < 0.001) and between BVL and MJD (*p* < 0.01) groups, and in ECF between Control and BVL (*p* < 0.001) and between Control and MJD (*p* < 0.001) groups. The post-hoc test showed that Controls [*F*(3, 57) = 25.7, *p* < 0.001], BVL [*F*(1.68, 13.47) = 4.19, *p* < 0.05], and MJD [*F*(2.07, 18.6) = 15.89, *p* < 0.001] groups had significant differences between conditions within each group.

### Spatial orientation tests

OPt showed significant differences between populations [*F*(2, 54) = 13.28, *p* < 0.001] with both BVL and MJD having larger deviation from the correct angle compared to Controls (*p* < 0.01 for BVL and *p* < 0.001 for MJD). No difference was found between BVL and MJD groups (*p* = 1) (see Table 2). Comparison within the BVL group of patients with and without oscillopsia found no significant differences between the groups in deviation from the correct angle (*p* > 0.05).

**Table 2:** Spatial orientation.

	OPt (deg)	SR (#)
Control	35.7 ± 24	6.86 ± 1.7
MJD	77.0 ± 25	5.05 ± 2.0
pBVL	68.9 ± 34	6.20 ± 1.6

Data represent mean ± STD.

Significant differences were found in the SRt test between populations [*F*(2, 54) = 5.48, *p* < 0.01], with a higher number of right answers found in Control vs. MJD (*p* < 0.01), but no difference was found between Control and BVL (*p* = 0.82) or between MJD and BVL (*p* = 0.19) groups as shown in Table 2.

Comparison within the BVL group of patients with and without oscillopsia found no significant differences between right answers in the SRt (*p* > 0.05).

**Figure 7:**
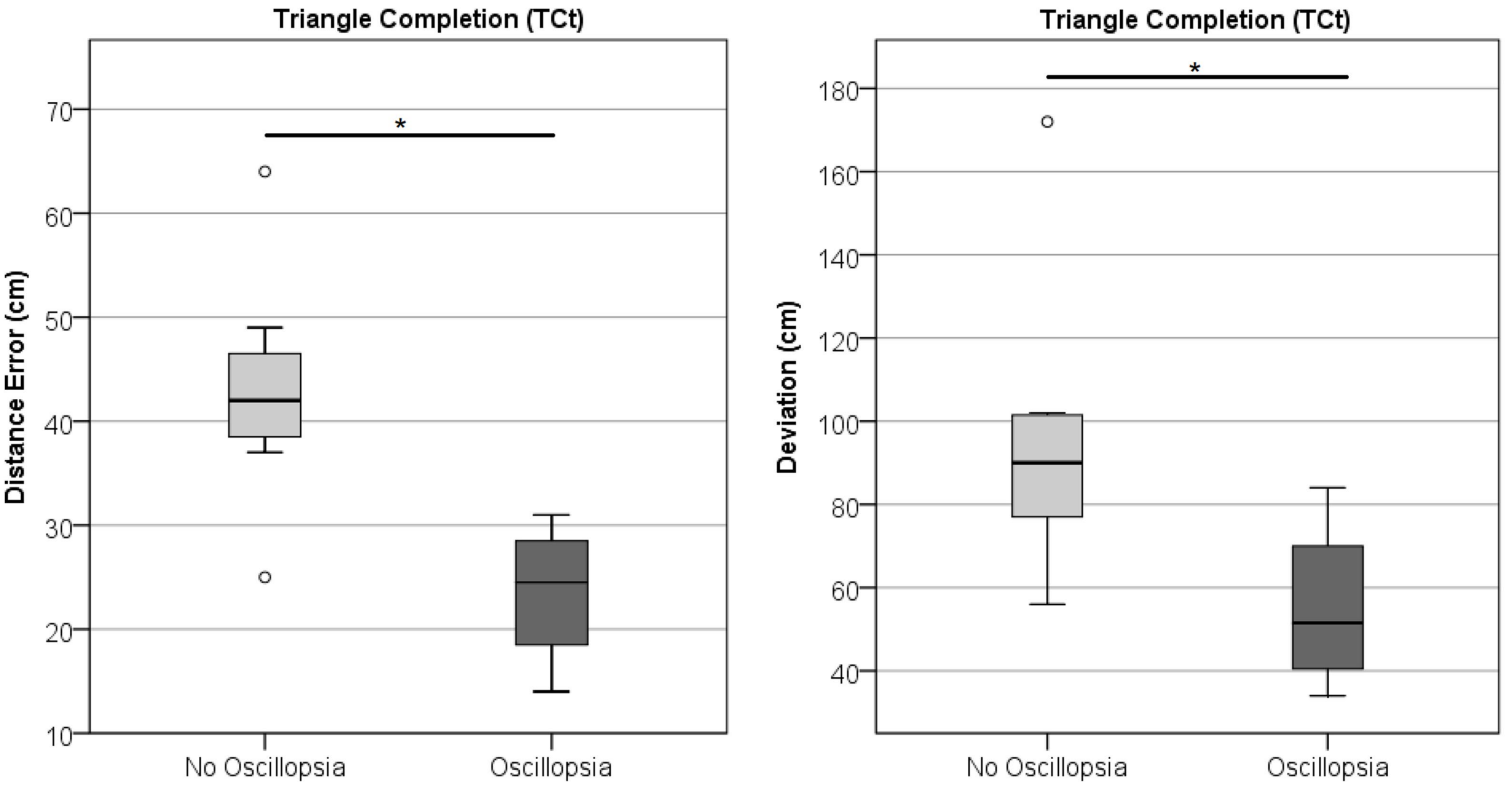
Triangle completion task. Distance and deviation between the BVL group without and with oscillopsia.

Neither Angle, Distance, nor Deviation showed differences between groups in the TCt (Wilks’ Lambda = 1.71, *p* = 0.132; BVL *n* = 11, MJD *n* = 9). Comparison within the BVL group of patients with and without oscillopsia (*n* = 4/7, respectively) found that the oscillopsia group demonstrated significantly less Distance (U = 2, Z = −2.27, *p* < 0.05) and Deviation (U = 3.5, Z = −1.99, *p* < 0.05) errors compared to the no-oscillopsia group as presented in Figure 7. Deviation and Distance were significantly highly correlated (*r* = 0.78, *p* = 0.005). No significant differences were found between groups in Angle errors (*p* = 0.11).

## Discussion

This study deals with an extensive evaluation of the relationship between bilateral vestibular hypofunction and various vestibular, saccades, balance, dizziness, and spatial orientation measurements together with an attempt to explain the complaint of oscillopsia. The first part of this discussion deals with the comparison between pBVL, MJD, and Control, whereas the second part of this discussion is focused on the differences between the oscillopsia and no-oscillopsia subjects in the pBVL population.

### Comparison between groups

As expected, the degree of VOR hypofunction and VOR remaining function was found to be significantly different between the impaired populations (pBVL and MJD) and the Control group. The impairment in pBVL was worse than that in the MJD group.

The perception of the subjective vertical in BVL has been reported to be altered when analyzing the absolute value of the deviation (29). However, in our study, no differences were found between groups for SVV nor dynSVV possibly due to our population being in a stable chronic stage. As expected, the moving background generated a greater conflict and dynSVV deviation was significantly higher than SVV for both CW and CCW background directions.

Saccadic eye movements in MJD have been reported to be impaired showing a certain degree of overshoot (30). In our MJD sample, we observed both overshoot and undershoot making the error results unreliable. For that reason, we calculated a new error value as the absolute value of the difference between the stimulus amplitude and the saccade amplitude (see Methods). Using this calculation, we found significant differences in the Accuracy error between the MJD and both Control and BVL groups. An intact cerebellum is needed for proper eye motor control with the dorsal vermis and posterior fastigial nucleus being especially important in the control of the saccades (31). Both areas were found to be severely affected in a pathoanatomical study in an MJD population (32), which can explain the impaired saccades, particularly the Accuracy error found in this study.

The posturography analysis demonstrated that both the BVL and MJD groups had significantly worse performance when eyes are closed compared to the Control group, supporting that visual information is an important source of balance information. With eyes open, the Control and BVL groups showed no differences in stability score, suggesting that the BVL population can compensate for balance impairments due to vestibular loss with visual information. Finally, the MJD group exhibited a much lower performance than Controls on all conditions, most likely related to their ataxia. While a clinical Romberg test is normal in BVL (33), a positive Romberg test on foam together with a positive head impulse test and reduced DVA has been suggested as a diagnosis test for BVL (34). Our results suggest that a positive Romberg test on foam may be further quantified by the stability score in computerized posturography.

The perception of space and spatial abilities also showed differences among our populations. Object perspective-taking is affected in both the BVL and MJD groups, while in the SRt, only the MJD group showed a significantly lower performance. This phenomenon suggests that while in the BVL group the spatial orientation is selectively impaired (self-rotation vs. object rotation), the MJD group appears to have a more generalized disability. Interestingly, no differences were found in TCT among the populations. It should be noted that although some studies exhibited differences in Angle among older adults in pBVL and Control groups (35), other studies have shown a poor reliability of this measure (36).

### Oscillopsia

One of the main difficulties when studying oscillopsia is that most of the published data do not clearly differentiate between acute and chronic or long-lasting oscillopsia. Acute oscillopsia is quite often in the BVL group, for example, due to antibiotic treatment ototoxicity and associated with the VOR hypofunction. However, VOR gain does not usually recover (37) and most patients with BVL do not have complaint of oscillopsia weeks or months after the oscillopsia started. Nevertheless, a portion of those BVLs never achieve compensation and experience chronic or long-lasting oscillopsia, even 20 years after the acute event. Both patient cohorts in this study had the VOR impairment at least for 9 months; therefore, the current study deals with chronic oscillopsia exclusively.

Although there is a significant VOR gain impairment in some subjects with MJD having less VOR gain than pBVL with reported oscillopsia, no individuals with MJD reported experiencing oscillopsia. We suggest that oscillopsia requires a VOR loss of peripheral and not central origin or a sudden and not progressive loss. The lack of oscillopsia in MJD subjects, together with already published data from other groups (38, 39), and our results on vHIT and SHIMP confirm the counterintuitive fact that chronic oscillopsia is not related to the degree of VOR impairment or VOR remaining function. As no difference was found between oscillopsia and no oscillopsia in the BVL group for the presence of cVEMP, oscillopsia appears not to be related to the saccular function as well.

The main finding of this study (although on a relatively small population) is that significant differences were found in various tests between BVL patients with and without oscillopsia. The importance of finding objective differences relies on the fact that some studies suggest that adaptation to oscillopsia is at least partially related to the patient’s frame of mind (5). Finding objective differences will help to understand the causes of oscillopsia and develop coping strategies to overcome it. The main complaint in patients with oscillopsia is that vision becomes blurred when moving the head and is manifested in the difficulty of recognizing faces or reading signs while walking. This subjective complaint is backed up by the DVA test conducted. Oscillopsia patients have a significant visual acuity reduction with head movements, supporting the idea that a real impairment and not only a purely subjective complaint is affecting these patients.

When the VOR is impaired, there is a need to recruit other eye movement strategies to compensate for the lost function. Catch-up saccades are used to compensate for reduced VOR and are related to the severity of the hypofunction (40). Even though saccade type was found to have no association with physical function in BVL (41), recent studies on saccades and oscillopsia suggest that saccades characteristics do play an important role in oscillopsia. The PR score (a measurement of saccades synchronization in vHIT and SHIMP tests) has been suggested as a measurement of compensation in BVL after finding that patients with higher PR values (non-synchronized saccades) in vHIT were more prone to oscillopsia (4). Our results are not in agreement with these findings, but the PR score was calculated in both studies with different tools that may have different criteria to determine saccades. Saccade patterns in SHIMP, particularly the presence of an “inappropriate” covert compensatory saccade during the head impulse, followed by a large anti-compensatory saccade, have been suggested as an indicator of a compensatory strategy. This saccade pattern may be evidence of how BVL patients have learned to trigger covert saccades during head movements and was also associated with a reduction of oscillopsia as compared to BVL subjects that do not present this saccadic strategy (42). Our BVL population did not show consistent behavior regarding the “inappropriate” saccades with all except one subject not even presenting covert saccades. The overt saccades observed in SHIMP may probably be attributed to correcting the gaze displacement due to the remaining vestibular function.

Our results showed that reflexive saccades latency with head still was different between the oscillopsia and no-oscillopsia subgroups. Latency of the reflexive saccade was found to be significantly different and an excellent classifier of BVL patients with and without oscillopsia. Contrary to what we expected, subjects with oscillopsia had saccade latencies that were comparable to Controls, while subjects without oscillopsia had longer latencies. It is interesting to note that these longer latencies are comparable to those in the MJD population. Although the delayed saccades latency was observed in a head still condition, a delayed saccade in real-life situations with head movements would produce a certain added amount of retinal slip and a delay in the adjustment for the retinal slip, so we would have expected this to be a contribution to oscillopsia. However, there is evidence that a negative correlation exists between oscillopsia and retinal slip (5). We suggest that this retinal slip may be providing a velocity signal of the head movement and at least partially compensating for the dysfunctional VOR in the programming and execution of the saccades to avoid visual instability and oscillopsia. This finding suggests that a compensatory mechanism responsible for recovering from oscillopsia comes at the expense of saccade latency. Further studies are required to explore this hypothesis.

The last test in which significant differences were found between subjects with and without oscillopsia was the TCt. This test evaluates one of the spatial orientation abilities related to the perception of space. BVL subjects have impaired spatial orientation (43) and are known to have hippocampus atrophy (44), which may at least partially explain this impairment (45). However, this is the first time that specific spatial abilities are tested between BVL with and without oscillopsia. Angle is the only parameter studied that is directly related to their impaired vestibular system. The first step toward returning to the starting point is to perform a rotation that will position the subject facing straight ahead of the endpoint. This parameter showed no significant difference between both groups, suggesting that both have a similar degree of difficulty in spatial orientation when a rotation is involved. Deviation and Distance were both significantly different between groups, and their high correlation is probably suggesting that Deviation differences are mainly because of the distance traveled and that taking the absolute value in each trial did not shadow any particular behavior. For this reason, this finding is very interesting and shows that BVL subjects with oscillopsia actually perform better than those without oscillopsia. We may hypothesize that as the oscillopsia population cannot truly rely on visual information and mostly relies on somatosensory and locomotor cues, when a challenge is presented that does not involve visual input, they perform better than the no-oscillopsia group, which, in daily life, lean on visual information to overcome the vestibular deficit.

In summary, four key findings are present in this study. First, the fact that oscillopsia was present in the pBVL group but not in MJD although the VOR gain is similar in both groups. Second, DVA was found to be different between pBVL with and without oscillopsia, showing that oscillopsia is not a subjective, perceptual symptom but a real impairment that decreases visual acuity, particularly when the head moves. Third, reflexive saccades are delayed in pBVL without oscillopsia compared to pBVL with oscillopsia (the latter even having similar values to Controls), which suggests that whatever compensation mechanism is being used to cope with oscillopsia, is reflected in the head still reflexive saccade latency and may be related to a velocity signal derived from retinal slip. Finally, spatial perception is worse in pBVL without oscillopsia as compared to pBVL with oscillopsia, probably because those with oscillopsia already rely less on visual input, suppressing that the visual input has less impact on their ability to navigate and orient their body in the world.

## Data availability statement

The raw data supporting the conclusions of this article will be made available by the authors, without undue reservation.

## Ethics statement

The studies involving humans were approved by Meir Hospital, Israel and Tel Aviv University, Israel. The studies were conducted in accordance with the local legislation and institutional requirements. The participants provided their written informed consent to participate in this study.

## Author contributions

DG: Conceptualization, Data curation, Formal analysis, Investigation, Methodology, Writing – original draft, Writing – review & editing. ZE: Conceptualization, Data curation, Investigation, Methodology, Writing – review & editing. RZ: Investigation, Writing – review & editing. MM: Conceptualization, Supervision, Writing – review & editing. CG: Conceptualization, Investigation, Supervision, Writing – review & editing.

## Footnotes

^1^Otometrics. ICS Impulse USB. User Guide. 2015.

^2^ https://www.imperial.ac.uk/brain-sciences/research/neurology/vestibular-neurology/

^3^123test (n.d.). Spatial reasoning test.
